# “Reclaiming the Narrative” of Sexual Violence Disclosures on Social Media: Social Reactions, Gender Norms, and Recovery

**DOI:** 10.1177/08862605261458221

**Published:** 2026-06-29

**Authors:** Svava Guðrún Helgadóttir, Erla Katrín Jónsdóttir, Alda Dís Arnardóttir, Karen Birna Thorvaldsdottir, Bryndis Bjork Asgeirsdottir, Rannveig Sigurvinsdóttir

**Affiliations:** 1 Reykjavík University, Iceland

**Keywords:** sexual violence, social media disclosure, recovery, social reactions, sex differences

## Abstract

In recent years, social media has become an important platform for sexual violence survivors to disclose their experiences and seek support. Since most research has focused on social reactions to in-person disclosures, less is known about social reactions in online spaces and how they relate to survivors’ mental health. Therefore, this qualitative study aimed to explore the aftermath of social media disclosures, focusing on the social reactions they evoke from others and how survivors perceive these as related to their recovery and well-being. A secondary aim was to explore potential sex differences in disclosures and social reactions, which remains a relatively unexplored area. In-depth interviews were conducted with 12 sexual violence survivors, 9 women, and 3 men, and the data were analyzed using reflexive thematic analysis. Five themes were developed: positive social reactions, healing, negative social reactions, losing control of the narrative, and setbacks in recovery. While all participants articulated experiencing validating and affirming responses that facilitated their recovery, many also described negative social reactions, including disbelief, rejection, and gossip, which hindered their healing process. Gender norms significantly influenced social reactions, with male survivors often facing skepticism and minimization rooted in societal expectations around masculinity. The findings highlight the dual role of social media as a source of empowerment and vulnerability. Furthermore, participants described healing after sexual victimization as a relational, complex, and nonlinear journey, emphasizing the importance of fostering an empathetic and inclusive environment that validates survivors’ voices and actively supports their healing.

## Public Significance Statement

This study shows that sharing experiences of sexual violence on social media not only can help survivors feel supported and aid in their healing, but it can also lead to harmful reactions like disbelief or rejection. The findings highlight the importance of fostering respectful, validating online environments, especially for male survivors, who may face added stigma due to gender norms.

Sexual violence is a serious global health issue affecting individuals across all genders and backgrounds worldwide (Borumandnia et al., 2020). Sexual violence includes various forms such as childhood sexual abuse, sexual assault during adolescence and adulthood, and sexual violence within intimate partner relationships, which can vary in severity, context, and psychological effects (Black et al., 2011; Dworkin et al., 2017). While research has often focused on women and children, there is a growing recognition that individuals of other genders are also affected by sexual violence (Easton, 2013; Pilkington et al., 2025). Globally, approximately 1 in every 5 women and 1 in every 14 men have experienced rape (Smith et al., 2018), the most severe form of sexual violence. Research further indicated that sexual victimization patterns differ by age and gender: Women experience higher overall rates across their lifespan, with elevated risk during adolescence and adulthood, whereas a relatively greater share of male survivors report their initial experiences of sexual victimization occurring in childhood rather than later in life (Cagney et al., 2025; Le et al., 2024).

The negative psychological and physical health consequences of sexual violence, such as anxiety, depression, trauma-related disorders, addiction, and self-harm, are well-documented (Bentivegna & Patalay, 2022; Quarshie, 2021; Sigurvinsdóttir et al., 2020). These adverse mental health effects may be further intensified by the decision to delay or refrain from disclosing those experiences (Ahrens et al., 2010).

### Sexual Violence Disclosures

As outlined in the Disclosure Process Model (2011), disclosure of sexual violence is an incredibly complex phenomenon, shaped both by what survivors hope to gain from disclosing (antecedent goals), by the disclosure event itself and immediate reactions to it, how they impact other concepts such as social support, as well as long-term outcomes for survivors. Most importantly, disclosure is not a single, linear process but can be ongoing as survivors decide to open up to different people with varying levels of detail (Chaudoir & Fisher, 2010). Disclosure also serves not only to communicate with others but also to help survivors understand and organize their thoughts about the event, enabling them to construct a more precise and structured narrative of their experiences (Starzynski et al., 2005). Despite growing awareness, sexual violence survivors commonly delay their disclosures for years, and unfortunately, some never disclose (Ullman et al., 2020). This raises concerns, as delaying disclosure may leave survivors facing serious consequences without necessary support (Ahrens et al., 2010; Alaggia & Wang, 2020).

There are various barriers to disclosures of sexual victimization. These barriers encompass internal factors such as shame and self-blame; interpersonal barriers such as power dynamics and fear of negative social reactions; and sociocultural barriers such as stigmatization and societal stereotypes (Collin-Vézina et al., 2015; Pijlman et al., 2023). Social reactions following disclosure, whether positive or negative, can significantly influence survivors’ thought processes, mental health, and willingness to seek further help (Bonnan-White et al., 2018; Dworkin et al., 2019; Jónsdóttir et al., 2024; Relyea & Ullman, 2015). Moreover, societal expectations and gender norms can influence the disclosure of sexual violence and subsequent social reactions, with men often being at a disadvantage (Okur et al., 2020; Weare et al., 2024).

### Social Media Disclosures and Reactions

Online spaces and digital interactions should be considered not as separate from the “real” world, but as an extension of it, continually intersecting with it. It can therefore extend and complement other types of communication, between specific members of networks of people, according to Rogers’s (2013) theory of digital methodology. As a result, new and interesting ways of disclosing sexual violence have opened up to survivors in recent years (Bogen et al., 2021). In 2017, many individuals used the hashtag #MeToo on Twitter to come together and form a supportive community centered around sexual violence disclosures (Alaggia & Wang, 2020). The MeToo movement and related initiatives have empowered survivors to break their silence openly (Gjika & Marganski, 2020) and played a crucial role in addressing sexual violence by providing survivors with a platform to share their stories and connect with a community (Mendes et al., 2019; Sigurvinsdóttir et al., 2019).

Online platforms can protect survivors from uncomfortable social reactions to their disclosures, allowing them to avoid witnessing a support provider’s nonverbal responses, such as crying, anger, or disgust (Bogen et al., 2021). Furthermore, online disclosures elicit more positive reactions than in-person disclosures (Bogen et al., 2019; Sigurvinsdóttir et al., 2019). Positive social reactions can significantly benefit survivors by reducing isolation and providing essential support, which in turn is related to improved mental health outcomes (Edwards & Ullman, 2018; Sylaska & Edwards, 2014). For instance, Bogen et al. (2019) analyzed tweets using the hashtag #NotOkay, where women shared their sexual violence experiences. Most tweets reflected positive social reactions, including advocacy for survivors, emotional support, tangible information, validation, belief in survivors’ experiences, and societal responsibility for change. Another qualitative study found that online respondents typically offered validation and defense for survivors, which contributed to improved mental health by reducing shame and fostering acceptance. Those who disclosed their experiences online often became advocates for other survivors, challenging victim-blaming narratives and educating their community (Gundersen & Zaleski, 2021). Similarly, Gueta et al. (2020) found that online disclosure was often driven by a need to find meaning and a commitment to advocate for a societal change, and a sense of resilient, activist identity facilitated survivors’ healing and recovery.

As with other types of disclosure, online disclosures can also have detrimental effects. For instance, a study analyzing 4,239 comments on news articles about sexual assault showed that 26% of the comments engaged in victim-blaming behaviors. These behaviors included questioning survivors’ credibility, suggesting hidden agendas, or blaming them for alcohol or drug use (Zaleski et al., 2016). Negative social reactions disregard survivors’ needs for support, thereby diminishing the positive effects of disclosures (Ahrens et al., 2010; Ullman & Najdowski, 2011). Experiencing negative social reactions is associated with increased severity of posttraumatic stress disorder symptoms, substance use disorder, depression, and other psychopathologies (Dworkin et al., 2019; Morris & Quevillon, 2021). Therefore, disclosure can, in some cases, be revictimizing, jeopardizing survivors’ sense of security, and reinforcing victimhood as a defining aspect of survivors’ identity (Gueta et al., 2020).

### Sex Differences in Online Reactions to Disclosure

Although further research is warranted, existing literature suggests that normative gender practices, particularly those related to societal expectations of masculinity, can significantly impact the dynamics of sexual violence disclosures (Easton et al., 2014; Sivagurunathan et al., 2019). Men who disclose instances of sexual violence in person generally receive less positive social reactions than women (Ullman & Filipas, 2005). Similar patterns have been observed with online disclosures by male survivors, who may receive comments that question their masculinity, minimize their experiences, or deny their victimization altogether (Loxton & Groves, 2022). In addition, sexual offending by women is often minimized or seen as unusual, which may further weaken the perceived legitimacy of victimization—especially for male victims (Wijkman et al., 2010).

However, despite increasing recognition of male sexual victimization, there remains a paucity of research addressing the motivations for disclosure among men, the reactions they receive, and the subsequent effects on their recovery processes (Pilkington et al., 2025). Recent qualitative research on men’s online disclosures indicates that public disclosure can both challenge silencing and expose men to vulnerability as they navigate concerns about legitimacy and gendered expectations of masculinity (Gueta et al., 2025).

Previous qualitative research has highlighted that online sexual violence disclosures can have both empowering and harmful effects. However, there is limited understanding of how survivors experience, navigate, and transition between these outcomes over time, especially on public social media platforms. Most studies categorize disclosure outcomes as either supportive or damaging, but less attention has been paid to how supportive and harmful reactions might co-occur or evolve during the same disclosure process. This study explores survivors’ experiences of social media disclosure, emphasizing how others’ reactions are interpreted within the framework of ongoing recovery in a gendered and culturally specific context.

### Current Study

Prior research highlights the psychological impacts of sexual violence and emphasizes the importance of social support following disclosure (Edwards & Ullman, 2018). However, gaps remain in understanding how these processes unfold in online environments, particularly across genders (Sigurvinsdóttir et al., 2019; Ullman & Filipas, 2005). A recent systematic review showed that most studies on online disclosures of sexual victimization were conducted in English-speaking countries, with predominantly female participants. Moreover, most relied on content analysis, which considers only the content of online posts without examining their impact on well-being. The authors concluded that the effects of online disclosures and social reactions to such disclosure remain largely unknown (Gorissen et al., 2023). Given the profound physical, psychological, and social impacts of sexual violence, along with the complex dynamics surrounding disclosures, it is essential to understand how social media shapes the experience of survivors around disclosures and reactions as well as their impact on well-being (Bonnan-White et al., 2018; Lorenz et al., 2018). While social media platforms provide new avenues for disclosing victimization, they also present challenges, including potential exposure to re-victimization caused by negative social reactions (Gueta et al., 2020). This study was conducted in Iceland, which has a population of around 400,000, and as one of the Nordic countries, Iceland is a technologically advanced society, where digital technologies are an important part of everyday life (Eurostat, 2021; Flower, 2024; Hägglund et al., 2023; Nesse & Erdal, 2022). Iceland ranks highly on international measures of gender equality (World Economic Forum, 2025) but still reports high rates of violence, which has been termed the “Nordic paradox” (Gracia & Merlo, 2016; Wemrell et al., 2019). As with other parts of the world, Nordic survivors often face victim-blaming attitudes and rape myths when they disclose violence (Bendixen et al., 2014). Some of the findings reported in this study could therefore easily be found in other parts of the world, but some, of course, could remain more specific to the local context. Therefore, the present study explores survivors’ experiences with social media disclosures, subsequent social reactions, and their impact on well-being and recovery, as well as the influence of gender norms.

This will be examined as part of the lived experience of Icelandic sexual violence survivors, with a focus on how public disclosure and social reactions affect their well-being and recovery. Building on the existing literature, this study aims to answer the following questions: (1) How do survivors experience social reactions to their disclosure of sexual violence on social media platforms? (2) How do survivors describe the effects of those reactions on their well-being and recovery? (3) How do survivors experience the influence of societal expectations and gender norms after disclosing on social media? These findings could inform the development of interventions and support tailored to the needs of survivors throughout their recovery and healing journey. A qualitative approach is well-suited to gaining an in-depth understanding of these complex and personal experiences.

## Method

### Participants

The study involved 12 participants aged 25 to 49 years (*M* = 33.33, *SD* = 5.85) who had shared their experiences of sexual violence on social media platforms (Table 1). All participants were Icelandic, with 10 identifying as female and 3 as male. The selection criteria required participants to be at least 18 years old and fluent in Icelandic. The time since participants disclosed their experiences on social media platforms ranged from 2 to 22 years (*M* = 7.71, *SD* = 6.24). They had encountered various forms of sexual violence, such as childhood sexual abuse, rape, sexual violence within relationships, and other forms of sexual misconduct, including sexual harassment, coercion, and unwanted sexual contact.

**Table 1.** Participants’ Characteristics and Social Media Platforms Used for Disclosure.

Pseudonym	Age at time of interview	Gender	Social media platforms used for disclosure	Time since disclosure
Tiffany	30 years	Female	Facebook profile and the traditional media	10 years
Jolene	33 years	Female	Statement on Facebook profile and a comment on TikTok	8 years
Veronica	31 years	Female	Twitter	5 years
Georgia	34 years	Female	Anonymously on an Icelandic page that mimics Reddit	21–22 years
Serena	31 years	Female	Facebook profile	8 years
Matthias	30 years	Male	Twitter	4 years
Irena	34 years	Female	Personal blog, traditional media, and interviews shared on Facebook profile	17 years
Gabriel	38 years	Male	Anonymously on a closed Facebook group and a press interview shared on Facebook profile	2 years
Thomas	31 years	Male	Anonymously on a closed Facebook group and on Twitter	2 years
Helena	49 years	Female	Closed Facebook group	10 years
Eve	34 years	Female	Facebook profile	3 years
Isabella	25 years	Female	Instagram	2 years

Several participants reported experiencing rape or childhood sexual abuse. Others described incidents of sexual violence within intimate or family relationships, and some reported mainly experiencing sexual harassment, coercion, or unwanted sexual contact. Many participants had faced multiple forms of sexual victimization throughout their lives. Participants used various social media platforms to share their experiences, with some disclosing more than once. Additionally, four participants initially chose to share their experiences of sexual violence on social media anonymously, and two of them later opted to disclose using their real names. All participants were identifiable to the researchers, and eligibility was verified through direct contact via email or direct message prior to participation. Eligibility was confirmed by verifying that participants met the inclusion criteria, disclosed sexual violence on social media, and by collecting contact details for scheduling the interview and obtaining consent procedures. Media exposure happened when participants disclosed their real names, and their posts attracted notable public interest. In such cases, journalists either reached out to participants directly or shared their posts via news outlets and social media platforms.

The concept of information power guided the determination of the sample size (Malterud et al., 2016), indicating that the more relevant information a sample provides to the study goal, the fewer participants are required. Factors such as the study’s aim, the sample characteristics, existing theories, interview quality, and analytical strategy influenced the adequacy of the sample size. Considering the specific study aim, the sample’s specificity, the rich information provided in the in-depth interviews, and the use of reflexive thematic analysis, a sample of 12 participants provided sufficient information power to draw meaningful and reliable conclusions.

### Procedure

The recruitment phase lasted from December 2023 to January 2025. Participants were selected using purposive sampling methods. Advertisements were posted on social media platforms, including Instagram and Facebook, as well as in organizations supporting survivors of violence, such as the Family Justice Center (Bjarkarhlíð) and an organization that supports survivors of sexual violence (Stígamót). Individuals could contact the researchers directly to express their interest in participating in the study. Six interviews were held in a designated meeting room at a university, one at a participant’s home, and six were conducted via Microsoft Teams. Before the interview, participants received written information about the study’s goals, procedures, confidentiality, and their right to withdraw at any time without consequences. At the start of each interview, informed consent was obtained either in writing, in person, verbally, or online, and participants confirmed their consent to audio recording. The duration of the interviews ranged from 32 to 120 min. The interviews were semi-structured and conducted by the first and third authors using an interview guide with 15 open-ended questions. These questions focused on participants’ experiences with disclosing sexual violence on social media, the social reactions they received, and their well-being following disclosure. Participants were also asked about previous in-person disclosures and associated social reactions as background context, but this information was reported only when directly relevant to social media disclosures, given the focus of the current study.

This study was approved by the *National Bioethics Committee* (*VSN-18-136*) and followed the APA ethical standards in conducting the study. Given the sensitive topic, participants could speak confidentially with a psychologist after the interviews if needed. No other compensation was offered for participation.

### Data Analysis

This study employed a qualitative approach involving semi-structured interviews that were conducted and analyzed using reflexive thematic analysis (Braun et al., 2022). Reflexive thematic analysis is a flexible method for analyzing qualitative data, which emphasizes the researcher’s active involvement in generating knowledge (Braun & Clarke, 2019). The researchers: (1) became familiar with the data, (2) generated initial codes, (3) collected codes to develop potential themes, (4) developed and reviewed the themes, (5) refined and defined the themes, and (6) wrote up the report (Braun & Clarke, 2006; Braun et al., 2022). An inductive approach was employed, with coding and theme development guided by the data, thereby capturing the rich, detailed nuances of the participants’ experiences (Thomas, 2006). Participants described various disclosure experiences throughout their lives, such as face-to-face disclosures to family, friends, or professionals. However, this study primarily focused on social media disclosures and their perceived consequences. Offline disclosures were treated as background context that influenced participants’ stories and interpretations of online sharing, rather than as a central analytic focus.

The first author coded the data and discussed potential themes with the research team. This thorough examination of the researcher’s interpretations constituted reflexive dialogue with research team members during the analysis phase, not during interactions with participants. Through this iterative process, codes were refined and grouped into five main themes. Theme boundaries were not seen as strict or mutually exclusive; instead, themes were viewed as interconnected processes that could occur together within participants’ experiences. In line with reflexive thematic analysis, inter-rater reliability was not calculated because coding is seen as an interpretive and reflexive process rather than a measure of coder agreement (Braun & Clarke, 2021). A thematic map was developed during the later stages of analysis to visually represent relationships among the themes as they were refined through ongoing coding and reflexive dialog. Coding and analysis were conducted using Atlas.ti (version 25; ATLAS.ti Scientific Software Development GmbH, 2023) qualitative analysis software.

As with all qualitative data analysis, the findings presented may reflect a subjective perspective. The authors of the study have a background in Psychology, with a specific focus on and interest in survivors of violence and their well-being following victimization. Most of the analysis was carried out by the first author, who is a clinical psychologist with experience working with survivors at a family justice center. This background may have shaped their sensitivity to specific themes in the narratives, potentially influencing how certain questions were framed and how the interviews were analyzed. However, experience in working with survivors may also have helped foster rapport, trust, and an empathetic, respectful interview environment with the study participants. Reflexivity was maintained through memo writing and team discussions, enabling ongoing reflection on assumptions, emotions, and decisions.

## Results

The findings reflect participants’ subjective interpretations of how social reactions following social media disclosure influenced their well-being and recovery.

**Figure 1.**
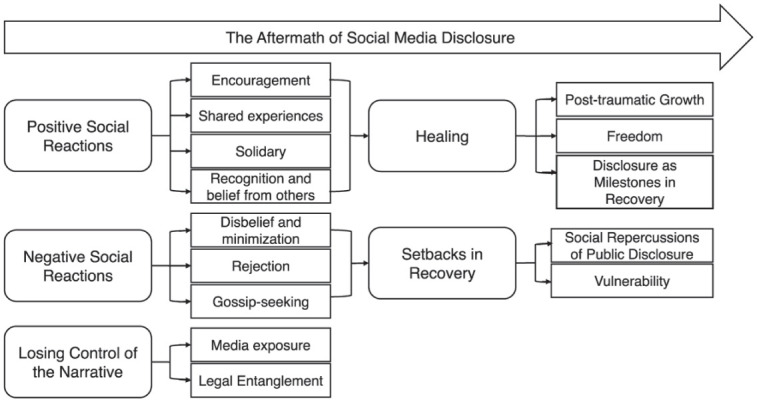
A thematic map of the aftermath of social media disclosures of sexual violence.

Figure 1 illustrates the complex aftermath of social media disclosures, characterized by diverse social reactions and varying levels of narrative control that impact survivors’ healing journeys. Importantly, the themes presented are neither mutually exclusive nor linear, as survivors often experience complex feelings following their disclosure that may evolve over time. Participants described shifting between feelings of healing and setbacks after the same disclosure, depending on the nature, timing, and social reactions. Positive and negative reactions often took place simultaneously, forcing survivors to constantly balance empowerment and vulnerability during recovery. The findings were divided into five main themes: (1) positive social reactions, (2) healing, (3) negative social reactions, (4) losing control of the narrative, and (5) setbacks in recovery. Positive social reactions facilitated healing, whereas negative social reactions were associated with setbacks in recovery. Each theme encompassed several sub-themes (Figure 1), described in detail below.

### Theme 1: Positive Social Reactions

Positive social reactions were a central theme observed in all participants’ narratives. This theme captures the varied, yet consistently positive responses participants received when disclosing their experiences of sexual violence on social media. The reactions were rooted in support-oriented behavior, fostering a therapeutic environment for survivors. Within this theme, four subthemes highlight the diverse aspects of these reactions: (1) encouragement, (2) shared experience, (3) solidarity, and (4) recognition and belief from others.

#### Encouragement

All participants encountered supportive and motivational feedback from their online community. For many, those reactions empowered them to continue their healing journey.

There was just so much praise for my bravery, for stepping forward and no longer hiding. It was primarily this courage that motivated me and significantly propelled me forward to achieve better recovery and gain a deeper understanding of my circumstances . . . enabling me to speak more openly about what I had experienced. (Jolene, female, 33 years old)

Although five participants felt afraid before disclosing on social media due to concerns about receiving negative feedback, they reflected on the relief they felt from the encouragement they received from the online community. For example, Matthias (male, 30 years old) shared: “*Fortunately, I received a lot of love and care from both those close to me and even from people I didn’t know at all. All that made me feel so good.*”

#### Shared Experiences

Five participants felt a sense of relief and belonging when they received reactions that made them aware they were not alone in their experiences. This understanding eased feelings of isolation and fostered a supportive community among participants and other survivors. Georgia (female, 34 years old) stated: “*And they just tell me, yes, I have been through the same, and they understand me and hope that I find someone to help me through this.*” Matthias (male, 30 years old) expressed similar sentiments when he said: “*It is good to feel that you can receive support from others who understand because people who have not experienced violence do not know what you are dealing with, and it is beneficial to feel that support.*”

#### Solidarity

Six participants discussed the importance of showing solidarity with other survivors by offering encouragement, understanding, strength, and support. They emphasized how they turned their painful experiences into a source of empowerment for others facing similar hardships. Encouraging fellow survivors to share their stories and receiving feedback from those who came forward because of them was profoundly meaningful.

I can’t count how many messages I received from other survivors, and initially, it was just a good feeling, then you think, yes, this matters to someone. And there were people of all ages, also older women, which perhaps surprised me the most. Women much older than me who might have been carrying something for many years, and then you get this feeling that it matters and that you are part of something bigger (. . .) the larger context involves countless victims who are shifting attitudes and the discussion. Yes, it felt good to be a part of that. (Tiffany, female, 30 years old)

Two participants recognized the broader influence of their actions, noting that their decision to pursue their sexual violence cases through the legal system and share their experiences on social media inspired others to do the same, as Irena (female, 34 years old) stated: “*I really appreciated hearing that girls were filing complaints, and you know, it felt like I was accomplishing something.*”

#### Recognition and Belief From Others

Eight survivors expressed the importance of being believed and acknowledged regarding their experiences of sexual violence. They emphasized the significance of validation from their community, friends, family, and others in their healing process. As Irena (female, 34 years old) stated, “*I was believed. I think that may have been the most important thing . . . There was never any doubt.*” A gender-specific pattern was especially noticeable among male participants, for whom validation was vital in confirming the legitimacy of their experiences with sexual violence. Specifically, two of the three male participants expressed a profound need for their experiences to be acknowledged as legitimate instances of sexual violence. Thomas (male, 31 years old) articulated how supportive reactions helped him understand that what he experienced was indeed violence. He stated: “*I had a tough time coming to terms with the fact that, you know, a woman could harm a man.*” He emphasized that validation from the online community was crucial in affirming his experiences, which challenged conventional gender dynamics, stating: “*A bit of validation for myself, you know. Or rather, a confirmation that it’s not just in my head, you know (. . .) I was pleased that I shared this, and I think that my only hesitation was just that I am a man.*” Gabriel (male, 38 years old) experienced similar emotions during the period when he held himself responsible for the victimization he suffered. His friend provided reassurance, affirming that he was not to blame: “*I was told, Gabriel, this is rape. This is pure rape. You shouldn’t blame yourself for this. Because I blamed myself, too (. . .) I blamed myself for not screaming, for not fighting back.*” These accounts show how masculinity norms influenced both initial self-doubt and the vital role of external validation in men’s recovery after disclosure.

### Theme 2: Healing

This theme shows how disclosing sexual violence on social media can help individuals process the associated trauma. All participants recognized that they continued to struggle with the harmful effects of sexual violence after their disclosure. Nevertheless, 11 felt that social media disclosure facilitated their healing journey. Positive reactions from others played a vital role in this process, encouraging survivors to manage their recovery more effectively. Disclosing their experiences online enhanced survivors’ sense of reclaiming control over their lives and narratives. The following three sub-themes illustrate the healing potential of online disclosures: (1) posttraumatic growth, (2) freedom, and (3) milestones in recovery.

#### Posttraumatic Growth

This subtheme offers insights into participants’ transformative journeys after sharing their stories on social media. Survivors reported personal growth, heightened resilience, and a renewed sense of empowerment following their disclosures. For instance, one participant redirected her post-disclosure energy toward actively supporting others who had faced similar experiences.

You can take something that is really terrible and has destroyed your life and do something positive with it. I think what was driving me forward in all of this was to take the emotions and, for example, also the anger, and use it as some force into something positive. (. . .) In my case, it was about creating content, writing a book, and creating campaigns to either demand something specific or raise public awareness. (Tiffany, female, 30 years old)

Jolene (female, 33 years old) shared how the positive reactions from others provided her with the courage to persist in her healing journey: “*It gave me a kind of purpose, (. . .) I am a bigger person than I present myself as. I have just as much right to be here as my abuser.*” Eve echoed similar sentiments: “*This gives people a platform to take back their, sort of . . . honor from the perpetrator, or you know, I own myself again.*”

#### Freedom

All participants discussed how sharing their experiences on social media gave them feelings of freedom and relief. Many shared how liberating it was not to carry the burden of trauma alone and how positive reactions empowered them to move forward. Participants also described the liberation from the weight of shame:

You kind of are . . . terribly much bottling it up inside, and often just with maybe all sorts of feelings and something uncomfortable that nobody knew I was going through. (. . .) I found it so good to just . . . everyone knew this, and you know, I didn’t need to be in hide-and-seek anymore. (Serena, female, 31 years old)

Others felt that disclosure helped them regain their autonomy from the abuser.

It would probably be the craziest feeling in the world when you start to experience this freedom (. . .) the abuser no longer has a hold on you, which naturally holds you back a bit, as it did for me initially in my recovery process. However, I can feel it, you know, that he’s not there anymore, he’s not right here by my ear, he has gone somewhere far away. (Jolene, female, 33 years old)

#### Disclosure as Milestones in Recovery

Nine participants believed disclosing on social media was a crucial milestone in their healing journey, representing significant progress for the survivors. For some, sharing their experience helped them find validation and clarity.

This was the final step in ensuring that it was no longer a secret because, you know, a secret is such a damn heavy burden in the backpack, so it’s nice to get rid of it even though there is still something left. (Veronica, female, 31 years old)

Other participants shared how reaching the milestone of disclosing their struggles on social media aided them in their journey to seek psychological help. Gabriel (male, 38 years old) said: “*You know, I had no other choice; it was really a question of whether I was going to work on myself or go down the path of losing myself.*” Others explained how sharing on social media helped them open up about their abuse in the first place and seek help.

Then I probably would never have told anyone, you know, and then it would just have been even more closed up inside me (. . .) It had a certain snowball effect on my life, to speak out, you know, yes, just having the courage to come out with it (. . .) I just needed to face reality and get back out into life and seek help and assistance and . . . fortunately, I did that. I would never be in the place I am today if I hadn’t had the sense to do so. (Matthias, male, 30 years old)

### Theme 3: Negative Social Reactions

About 9 out of 12 reported experiencing negative social reactions following their disclosures on social media, both in comments and in person. These reactions varied significantly among participants, highlighting the complexity of societal responses. Negative social reactions after disclosure included three subthemes: (1) disbelief and minimization, (2) rejection, and (3) gossip-seeking. Each subtheme presented a unique societal challenge that survivors had to navigate.

#### Disbelief and Minimization

Several participants encountered reactions that invalidated their experiences of sexual violence after their disclosures. One female participant described how the offender’s family reacted with intense denial, suggesting that she was infatuated with him. Among male participants, expectations of masculinity significantly influenced the negative social reactions they experienced. Two out of three men described how these norms affected the social responses they received. These expectations manifested in ways that were challenging to navigate, exacerbating feelings of shame and inadequacy among survivors. Specific reactions included intrusive questions about why they did not respond differently during the abuse, as Gabriel (male, 30 years old) shared: “*Men are just not believed, you know . . . As a man, why didn’t you hit him? Why didn’t you kill him? I mean, I understand women wouldn’t do that, but why didn’t you? You’re a man?*” These reactions reflect gendered expectations that men should be able to physically resist and should not be seen as sexually victimized. This can exacerbate shame and hinder recovery by framing survivors’ responses as personal failings instead of recognizing the assault. Thomas (male, 31 years old) expressed his feelings of humiliation when his male friend minimized his sexually abusive experience by adopting a dismissive attitude toward the incident: “*Yeah, maybe it’s not right, but I wouldn’t call it rape.*”

#### Rejection

Five participants faced rejection after sharing their experiences of sexual violence on social media. The nature of these rejections varied, both subtly and overtly. One participant discovered that his post was not accepted in a private group, deepening his sense of exclusion from a community he had hoped would provide support and understanding. Others experienced avoidance in their personal lives, as former friends, acquaintances, and even family members distanced themselves, refusing to engage in conversations or withdrawing from their relationships. Rejection also appeared as people in the participants’ social circles chose to side with the offender rather than support the survivor. For example, Irena (female, 34 years old) described how her family members reacted negatively toward her, framing the disclosure as an act that had harmed the family: “*And then somehow I just received a letter from them (. . .) [asking me] whether I didn’t realize what I was doing and everything with opening this up and it was me who was destroying the family*.”

Rejection was expressed not only through overt exclusion and distancing but also through subtler forms of withdrawal, such as silence and lack of acknowledgment. The absence of social responses to participants’ disclosures had significant effects on survivors’ recovery. While some participants faced explicit negative feedback, two reported experiencing silence from those they expected to support them, particularly family members. This lack of recognition often led survivors to feel overlooked and emotionally invalidated by those whose reactions they sought. The participants expressed disappointment that close relatives failed to acknowledge their disclosures, despite being aware of them. Gabriel (male, 30 years old) described: “*I think people tend to avoid talking about this (. . .) It’s more like there was some kind of wall (. . .) Men don’t talk about these things, I work in a male environment, no women are working with us.*” This lack of response intensified the emotional weight of the experience, underscoring that the absence of a reaction can be perceived as a form of rejection.

#### Gossip-Seeking

Three participants highlighted gossip-seeking behavior as a reaction that negatively affected their experiences with disclosure. This included probing questions about the perpetrators, the circumstances of the assault, and specific descriptions of the violence in offline and online spaces. They felt that specific individuals were more interested in the sensational aspects of their experiences with sexual violence than in showing empathy.

It was perhaps the only thing I found uncomfortable when I felt that people were going too far in asking for details, you know, asking more about the perpetrators, or the violence itself, you know, the actual descriptions of the violence (. . .) I found that uncomfortable. (Tiffany, female, 30 years old)

Other participants expressed unease upon learning that some individuals had contacted their families and friends to gather personal and sensitive information about their experiences. Moreover, one participant observed that personal messages often contained inappropriate curiosity. Jolene (female, 33 years old) noted: “*There were so many people that were like, OMG, I didn’t know this happened. Who was this?*” Across accounts, gossip-seeking was characterized by repeated or unsolicited requests for information that participants experienced as intrusive and misaligned with their needs at the time of disclosure.

### Theme 4: Losing Control of the Narrative

Losing control of the narrative was consistently experienced as a harmful consequence of social media disclosure. Participants described how increased public visibility reduced their ability to regulate who accessed, interpreted, or acted on their stories, thereby increasing the risk of misrepresentation, unwanted attention, and secondary victimization. Notably, these experiences were predominantly described by women in the sample. This diminished control contributed to setbacks in recovery and, in some cases, hindered healing despite receiving validating and supportive reactions.

Several participants encountered unintended consequences after sharing their experiences of sexual violence on social media. This theme was divided into two sub-themes: (1) media exposure and (2) legal entanglement, each emphasizing how survivors’ control over their narrative was undermined following their disclosure.

#### Media Exposure

This subtheme illustrates how participants’ disclosures can quickly reach audiences beyond their initial intent. Four participants described how media exposure can lead to widespread sharing of participants’ experiences, resulting in a loss of control over how accounts of sexual violence are represented and discussed. Extensive exposure may sometimes distort the original narrative, leading to misinterpretations that further victimize the survivor. Eve (female, 34 years old) expressed it this way: “*And then I was named in the media, and then more media began contacting me, and then suddenly it all started to become uncomfortable because this was not what I wanted. I did not want to become someone like that.*” Similarly, Irena (female, 34 years old) described how her blog post unexpectedly caught the media’s attention: “*And then I wrote a blog about my experience, and apparently someone sent it to the media, because I got a phone call from two of the major media outlets, which was really kind of strange (. . .), so it all felt very uncomfortable.*” This unexpected exposure contributed to a feeling of losing control over how her story was shared and who had access to it.

#### Legal Entanglement

This subtheme explores the legal complexities that can arise from a participant’s social media disclosures. After her disclosure, one participant, Helena (female, 49 years old), became embroiled in a defamation case when her offender attempted to sue her for defamation, which he ultimately lost at trial. She described the legal proceedings that followed as an overwhelming extension of her trauma, leading her to perceive the legal battles as a form of secondary victimization: “*This is really violence on top of violence, with the assistance of the legal system, legally.*” Helena conveyed that the legal repercussions following her disclosure significantly hindered her healing process, and if given the opportunity, she would have chosen not to disclose in that manner.

The recovery process and everything were set back 100%. You know, I didn’t gain anything from this (. . .) It cost me money, took a tremendous amount of time, and cost my family a whole lot. I would never do this again. (Helena, female, 49 years old)

### Theme 5: Setbacks in Recovery

This theme describes the challenges and complexities that survivors encounter in their healing process after disclosing sexual violence on social media. Although disclosure can facilitate paths to recovery, it may also lead to significant setbacks due to societal reactions and online interactions that survivors cannot control. Two subthemes: (1) social repercussions of public disclosure and (2) vulnerability, highlight the nature of these setbacks.

#### Social Repercussions of Public Disclosure

Four participants shared their experiences of societal repercussions following their disclosures on social media. In these cases, online disclosures reshaped how others perceived them, defining them exclusively through the lens of victimhood. This highlights how societal backlash and attitudes can exacerbate trauma, potentially leading to discrimination and exclusion for individuals who have experienced sexual violence. These societal reactions can marginalize survivors and discourage others from coming forward.

You don’t want to be known as just the girl who was raped; it is solely your story, what you are known for. You just don’t want it to be your identity, that people see you based on that. That’s what you don’t want. (Tiffany, female, 30 years old)

This concern extended beyond identity to anticipated real-world consequences, such as employment opportunities. As Tiffany further explained: “*I am fully aware that, for example, if I apply for a job, this is what might come up, and that it can affect how people perceive me. Sometimes it has even felt like I have been allowed less because people immediately think, she is struggling, you know, she was raped.*”

#### Vulnerability

Seven participants reported increased emotional and psychological exposure following their disclosures of victimization. Negative social reactions considerably impacted this vulnerability, affecting various areas of their lives. This vulnerability influenced their social interactions, self-esteem, and professional relationships:

About six months after the disclosure, I started to feel like, oh shoot . . . It was more like . . . I began having second thoughts because I was in a bubble where I didn’t want anyone to know who I was. I had had enough. (Eve, female, 34 years old)

This highlights the broader significance of post-disclosure vulnerability as survivors continue to navigate continual public and private reactions to their narratives. One participant expressed that revealing her experience of sexual violence in a formal public setting, such as the media, intensified her sense of vulnerability as she reflected on the potential long-term consequences of being publicly linked to her traumatic experiences.

I became very aware that this disclosure would be something that would follow me for the rest of my life . . . Stepping into the media spotlight makes it clear that these disclosures will last forever. I deeply considered whether I might regret this later, whether it could be used against me. (Tiffany, female, 30 years old)

## Discussion

This study explored the experiences of 12 survivors following social media disclosures of sexual violence, the social reactions these disclosures provoked, and their effects on well-being and recovery. Through reflexive thematic analysis of in-depth interviews, five main themes were developed: positive social reactions, healing, negative social reactions, losing control of the narrative, and setbacks in recovery. Much of the existing literature on social reactions to disclosures was developed prior to the rise of social media, resulting in a limited understanding of how such reactions unfold in online contexts (Sigurvinsdóttir et al., 2019). Therefore, the findings provide valuable insights into the dual role of social media, highlighting its potential as an empowering platform for survivors while presenting a challenging and emotionally vulnerable environment for some.

A central finding is that social reactions, whether affirming or invalidating, play a pivotal role in shaping the aftermath of disclosures. This aligns with previous literature emphasizing the importance of social reactions in shaping survivors’ psychological outcomes (Dworkin et al., 2019; Jónsdóttir et al., 2024). Supporting prior findings (Bogen et al., 2021; Gundersen & Zaleski, 2021), all participants described receiving some form of positive reactions following their disclosure on social media, such as encouragement, shared experiences, solidarity, as well as recognition and belief from others. Receiving empathy and validation acknowledged participants’ experiences and reduced feelings of shame, isolation, and self-blame. These findings underscore the relational nature of healing in online spaces and support prior research, which indicates that emotional support, belief, and empathy are crucial factors in survivors’ recovery (Bogen et al., 2019; Ullman, 2000). For some, positive social reactions prompted further steps in their recovery, such as seeking therapeutic help or engaging in advocacy. These reactions played a crucial role in the healing theme, promoting posttraumatic growth, a sense of freedom, and progress in participants’ recovery, aligning with earlier literature (Alaggia & Wang, 2020; Sigurvinsdóttir et al., 2019).

The power of shared experience and solidarity was prominent in participants’ accounts. Recognizing that others had endured similar experiences brought relief and diminished isolation. Moreover, participants emphasized the importance of providing solidarity to fellow survivors. Feeling less isolated through connection and reclaiming agency by supporting others appeared to mutually reinforce one another, highlighting the relational nature of healing within online spaces. Observing others disclose personal experiences on social media can serve as a powerful motivator for individuals to share their own. This dynamic may be particularly characteristic of the online context, where the public and visible nature of disclosures can foster a sense of solidarity and perceived safety (Sigurvinsdóttir et al., 2019). These findings echo previous research underscoring the power of a supportive environment and community following disclosure (Edwards & Ullman, 2018; Gueta et al., 2020; Sylaska & Edwards, 2014) and reinforce the idea that healing after trauma is not solely an individual process but is significantly shaped by relational dynamics (Alaggia & Wang, 2020).

Conversely, many participants encountered negative reactions after disclosing their experiences, including disbelief and minimization, rejection, and gossip-seeking. These reactions were painful and disrupted the healing process for some. Survivors’ experiences resonate with Ullman’s (2000) categories of negative social reactions, including victim-blaming and rejection. Negative social reactions led directly to setbacks in recovery, including social repercussions and a sense of vulnerability. These findings align with research linking negative reactions to increased distress and shame (Ahrens et al., 2010; Dworkin et al., 2019; Ullman & Najdowski, 2011), showing that they may serve as a form of revictimization (Gueta et al., 2020). Online disclosures more readily allow for social reactions from strangers (Sigurvinsdóttir et al., 2019), who may be more likely to respond negatively due to the anonymity and social distance afforded by digital platforms. These responses are of significant concern as they can have far-reaching effects, as witnessing negative feedback online may discourage others from disclosing their own experiences (PettyJohn et al., 2021).

The reactions reported by participants in this study somewhat overlap with quantitative studies on social reactions, but not fully. For example, the positive reactions of the social reactions framework (Ullman, 2000) include emotional support and tangible aid, which may relate to our interview theme “encouragement,” but does not include our other positive themes of “shared experiences,” “solidarity,” and “desiring recognition and belief from others.” Our themes, therefore, reflect that disclosures are a continually interactive process, which may involve the other person also sharing their own experiences of victimization. The negative social reactions framework consists of blame, control, distraction, egocentric responses, and treating the survivor differently than before (Ullman, 2000). These can be seen to relate to our interview themes of “disbelief and minimization” as well as “rejection.” However, the theme, “gossip-seeking,” seems to reflect different concepts. Although gossip-seeking may overlap conceptually with egocentric reactions, our findings suggest it reflects a more specific pattern characterized by curiosity-driven information seeking, including requests for details about the assault or the individuals involved. Notably, while Ullman (2000) acknowledged that egocentric responses can include curiosity about the assault, such behaviors are not explicitly operationalized within the SRQ. As such, gossip-seeking may represent an underexamined dimension of negative social reactions, particularly in contexts where disclosures reach broader, perhaps less personally connected, audiences, as with online disclosures. The fact that our interview themes overlap to a certain extent with the social reactions framework is not surprising, as the social reactions framework was developed to refer to in-person disclosures, not on social media. The current findings also reflect the strength of using in-depth interviews, which allow participants to fully explain the factors they see as influencing their disclosure and subsequent well-being.

Another noteworthy contribution of this study is the theme of losing control of the narrative, highlighting how disclosures that initially appear empowering can become overwhelming. Some participants faced unwanted media attention or legal repercussions, adding to their emotional strain and loss of autonomy following their disclosure. One participant’s legal defamation case significantly obstructed healing, illustrating how reactions to disclosure can exacerbate trauma instead of alleviating it. These findings echo prior research highlighting that online disclosures can spiral beyond survivors’ control, leaving them vulnerable to misinterpretation (Gorissen, 2024; Gueta et al., 2020). While many survivors turn to social media to reclaim their narrative (Alaggia & Wang, 2020), this ownership can be threatened when others distort disclosures (Gorissen, 2024). In such instances, speaking out may paradoxically lead to a renewed loss of narrative ownership. In some cases, this loss of control over the narrative and difficult feelings experienced as a result may even stem from well-meaning reactions from others. Further exploration of this matter is necessary to understand how social media platforms influence survivors’ ability to maintain ownership of their narratives.

A focus of this study was how gender norms and societal expectations shape the social reactions survivors receive. The findings support earlier research in several ways (Easton et al., 2014; Sivagurunathan et al., 2019). All three male participants experienced feeling the weight of traditional masculinity norms. Two experienced adverse social reactions rooted in gendered expectations, including disbelief, minimization, and questioning of their reactions during victimization. These reflect societal narratives that frame males as invulnerable, supporting earlier findings (Loxton & Groves, 2022; Pilkington et al., 2025; Weare et al., 2024).

These findings are consistent with recent qualitative research on men’s online disclosures of sexual violence (Gueta et al., 2025). This research highlights that public disclosure can challenge silencing but may also increase vulnerability to minimization and disbelief, influenced by masculinity norms. Similar to previous studies, male participants expressed concerns about legitimacy and gendered expectations regarding how men should respond. These perceptions influenced both the reactions they received and the emotional impact on their recovery. Gabriel expressed shame for not having fought back during the victimization. This feeling was intensified by assumptions from others about how he should have physically resisted. These results highlight the enduring impact of gender norms and stereotypes that may discourage male survivors from disclosing or seeking support following sexual violence (Easton et al., 2014).

Although the analysis was not designed to explicitly compare subgroups, participants’ responses indicated that social reactions and recovery processes are shaped by a combination of gender, relationship to the perpetrator, and the disclosure setting. Male participants more frequently discussed issues related to legitimacy and masculinity norms, while disclosures involving family abuse were often linked to increased fears of rejection and long-term relational impacts. Disclosing in highly public forums or using real names seemed to heighten the risk of losing control over the narrative, while disclosures in more closed or semi-private environments was usually perceived as emotionally safer, though with a more limited audience. Nonetheless, these patterns were not consistent, as many participants occupied multiple roles at once, underscoring the complexity and nonlinearity of their post-disclosure experiences.

While healing and setbacks in recovery emerged as distinct themes, they were closely tied to the nature of participants’ social reactions. In many cases, the same disclosure produced healing and setbacks, depending on who reacted and whether the reaction was positive or negative, as survivors typically receive different reactions from multiple sources (Jónsdóttir et al., 2024). While setbacks in recovery reflect survivors’ emotional and relational consequences following disclosure, losing control of the narrative illustrates the specific processes through which public disclosure caused renewed harm, such as media exposure and legal entanglements.

Consequently, participants may feel a sense of empowerment from the solidarity of online supporters, yet simultaneously experience rejection due to silence from their loved ones. This duality underscores the notion that recovery from trauma is seldom a linear process and online platforms can offer both opportunities and challenges, contributing to the fragility of survivors’ healing journeys (Gueta et al., 2020).

## Limitations

These findings provide valuable insights into participants’ lived experiences following social media disclosures, though some limitations must be acknowledged. The sample consisted solely of Icelandic individuals, including nine women and three men, most of whom were in their 30s. This cultural and demographic homogeneity may limit the transferability of the research findings to sexual violence survivors from other contexts, particularly those from racially, ethnically, or socioeconomically diverse communities, or societies with different gender norms and social support systems. For example, Iceland’s relatively small and homogeneous population, strong welfare infrastructure, high levels of social trust, and distinct gender-equality culture may shape survivors’ experiences in ways that are not directly generalizable to other settings.

The Icelandic context may have influenced participants’ experiences in specific ways. Iceland’s small population and close social networks can amplify both positive and negative social reactions following disclosure, raising risks like loss of anonymity, media coverage, and damage to reputation. These findings should be viewed within this specific sociocultural setting, and their applicability to other contexts should be assessed based on differences in population size, media landscape, and social norms.

Participants were recruited through social media and survivor-support organizations, resulting in a self-selected sample. As a result, the findings may mainly reflect survivors who felt willing and able to discuss their disclosure, potentially underrepresenting those who feared identification, avoided revisiting their experiences, or were less connected to survivor communities. This limitation is especially relevant for male survivors, as stigma and masculinity norms may further reduce men’s willingness to participate, so the men included might not represent the full range of male post-disclosure experiences.

While the small sample of 12 participants might be viewed as a limitation, extensive data were gathered from those participants, providing sufficient informational power. Recruiting male participants proved particularly challenging despite vast efforts, highlighting ongoing gender-based barriers in discussions about sexual violence (Borumandnia et al., 2020; Easton et al., 2014). Furthermore, future research should explicitly examine sexual orientation, gender identity, and other intersectional social identities, as these can influence stigma, safety, and responses to disclosure both online and offline settings. Social media recruitment may have influenced the results, reflecting a group of survivors more willing to share their experiences. This limits representativeness, although it also demonstrates an interest in articulating their experiences in rich detail, which can be considered a strength of the data.

## Future Research Directions

Future studies must incorporate a broader range of participants that reflect various gender identities, ages, cultural backgrounds, and other dimensions of diversity (e.g., socioeconomic status, ethnicity) to explore how social media disclosures unfold in different sociocultural settings and across individual characteristics. Moreover, comprehensive research into the societal dimensions of masculinity is essential for understanding the barriers men face in disclosing sexual violence and help-seeking. Further efforts are needed to explore sex differences in online disclosure, social reactions, and recovery trajectories, with particular emphasis on recruiting a sufficiently large and diverse sample of male survivors. Furthermore, longitudinal studies could provide valuable insights into how social reactions impact survivors’ healing and well-being over time. Ultimately, examining the effects of online anonymity and specific platform features may help identify factors that foster safer environments for disclosure.

## Clinical Implications

The findings hold significant value for mental health professionals, advocates, and policymakers working with survivors. Clinicians should recognize the multifaceted effects of online disclosure, noting its empowering and potentially distressing consequences. Supporting survivors in making informed disclosure decisions in advance and discussing potential risks and the emotional impact of receiving diverse social reactions can be essential. Media professionals are also responsible for reporting survivors’ disclosures with consent, empathy, and care, recognizing the lasting impact of narrative framing. Educating the public on empathetic and appropriate reactions to disclosures is crucial for building a safe in-person and online environment.

## Conclusion

This study emphasizes how social reactions on social media were perceived by participants to shape mental health and recovery following sexual violence disclosures among Icelandic participants. The five identified themes—positive social reactions, healing, negative social reactions, losing control of the narrative, and setbacks in recovery—highlight social media’s complex role in trauma recovery. Positive reactions, such as validation, shared experiences, and solidarity, contributed to healing and empowerment. Conversely, negative social reactions hindered recovery efforts. Notably, gender norms played a role in shaping the reactions received by male survivors, underscoring the necessity of confronting traditional masculinity stereotypes. While social media can offer empowering opportunities for survivors, it also poses emotional risks, reflecting the relational and dual nature of recovery in public digital spaces. This knowledge can support survivors in making informed decisions about disclosing their experiences online and highlights the importance of increasing public awareness of sexual violence against men to reduce stigma toward male survivors.
